# Genetic and regulatory architecture of meat yield traits in commercial pig populations

**DOI:** 10.1186/s12711-026-01104-2

**Published:** 2026-11-09

**Authors:** Yani Huang, Mingyang Wang, Mingfang Zhou, Shijun Xiao, Zhiyan Zhang

**Affiliations:** National Key Laboratory for Swine Genetic Improvement and Germplasm Innovation, Jiangxi Agricultural University, Nanchang, 330045 China

## Abstract

**Background:**

Lean meat percentage (LMP), longissimus muscle area (LMA), and longissimus muscle depth (LMD) are critical carcass traits that directly impact meat yield and the economic efficiency of swine production. Although genome-wide association studies (GWAS) have identified numerous loci associated with these traits, the functional characterization of candidate quantitative trait loci (QTLs) and their regulatory mechanisms is still limited and poses significant challenges. Here, we conducted GWAS in 4593 pigs from three commercial populations (Yorkshire, Landrace and Duroc) to identify loci associated with LMP, LMA and LMD. We further integrated integrative chromatin accessibility, histone modifications, chromatin interaction information, and dual-luciferase reporter assays to identify candidate QTLs with potential regulatory functions. Candidate genes were subsequently prioritized using single-nucleus RNA sequencing, Phenome-wide association analysis, and Mendelian randomization.

**Results:**

This multi-layered approach prioritized candidate regulatory QTLs associated with LMP, LMA and LMD, and identified high-confidence candidate genes potentially involved in the regulation of meat yield traits. For LMP, *WWOX* and *ABL1* showed the strongest evidence in Yorkshire and Landrace population, respectively. For LMA, *PHB1* and *BAZ2A* showed the strongest evidence in the Yorkshire and combined-population, respectively. *SLC16A7* was specifically associated with both LMP and LMA in Landrace. For LMD, *ENSSSCG00000045482* and *RSPO4* showed the strongest evidence in Yorkshire pigs, while *XPO4* was identified in the combined-population analysis. These genes may influence muscle metabolic homeostasis, muscle proliferation, hypertrophy, and atrophy through interactions with myogenic transcription factors and key signaling pathways such as PPAR-RXR and Wnt/β-catenin.

**Conclusions:**

These findings provide new insights into the genetic and regulatory basis of meat yield traits in pigs and highlight candidate regulatory QTLs and genes for future functional validation and precision breeding applications aimed at improving pork yield.

**Supplementary information:**

The online version contains supplementary material available at https://doi.org/10.1186/s12711-026-01104-2.

## Background

Pork is a major source of animal protein for human consumption in many countries worldwide. Improving pork yield and lean meat percentage (LMP) has long been a primary objective in pig breeding programs [1]. LMP, loin muscle area (LMA) and loin muscle depth (LMD) are key meat yield and carcass traits. Previous studies have shown that both LMA and LMD are strongly positively correlated with LMP, reflecting their shared biological basis in muscle development and carcass composition [2, 3]. Genome-wide association studies (GWAS) have successfully identified numerous loci associated with LMP, LMA, and LMD in different pig populations [4–7]. However, interpreting these associations in a biological and genomic mechanisms remains limited and challenging.

Recent advances in functional genomics have highlighted the critical roles of regulatory elements, including enhancers, promoters, and higher-order chromatin organization, in shaping complex phenotypes [8–10]. Integrating GWAS signals with epigenomic features and transcriptional regulation provides a powerful framework to move beyond statistical associations and toward biological interpretation. Nevertheless, for economically important meat yield traits in pigs, systematic integration of GWAS with tissue-relevant regulatory landscapes and causal inference remains limited. Given that LMP, LMA, and LMD are tightly linked to muscle and adipose tissues, we hypothesized that these tissues harbor specific epigenetic contexts that allow trait-associated SNPs to perturb gene expression related to muscle development and adipose deposition, thereby shaping phenotypic variation.

Duroc, Landrace, and Yorkshire pigs are globally recognized lean-type commercial breeds and constitute the genetic foundation of most intensive crossbreeding systems, accounting for a dominant share of the global pork industry [11]. In this study, we conducted GWAS for LMP, LMA, and LMD in 4593 pigs from these three commercial populations. By integrating chromatin accessibility, histone modifications, and chromatin interaction data from muscle and adipose tissue, we identified candidate regulatory QTLs with potential functional relevance. Candidate genes were further evaluated using single-nucleus RNA sequencing (snRNA-seq), Phenome-wide association analysis (PheWAS), and Mendelian randomization (MR) to provide complementary lines of evidence for candidate gene prioritization. Through this multi-layered approach, we aimed to identify candidate genes and putative regulatory mechanisms associated with meat yield traits, providing insights into the molecular basis of carcass composition and potential targets for precision breeding and meat yield improvement.

## Methods

### Animal and phenotype collection

A total of 4593 pigs born between May 2022 and March 2023, comprising 1563 boars and 3030 sows from three purebred American lines, Yorkshire (*n* = 2585), Landrace (*n* = 1290), and Duroc (*n* = 718), were included in this study. All animals were raised in the nucleus breeding herds at the same farm under standardized management and rearing conditions, following consistent breeding programs within each breed. The sampled individuals were from the same generation and were measured during the same sampling period. To increase the sample size, animals aged 136–217 days were included in the study. Body weight (BW) data were recorded, LMA (cm^2^), LMD (mm), and backfat thickness (BFT) were measured by B-mode ultrasonography in accordance with the NY/T 2894 guidelines. Ultrasound measurements were performed on the left side between the third and fourth ribs from the last rib. The transducer was placed parallel to the dorsal midline at approximately 5 cm lateral to the midline to obtain longitudinal images for LMD measurement, whereas a perpendicular orientation to the dorsal midline was used to acquire transverse images for LMA determination. LMP (%) was estimated based on ultrasound measurements of BFT and LMA, combined with BW, following standard commercial evaluation procedures. Specifically, LMP was calculated using the following equation:

1 $$y = 67.264 - 0.063{X_1} - 2.122{X_2} + 0.205{X_3},$$

where y represents estimated LMP, X_1_ is BW, X_2_ is BFT, X_3_ is LMA.

### Genotyping, imputation, and quality control

The genomic DNA was extracted from ear tissues of 4593 pigs with a standard phenol-chloroform method, and high-quality DNA samples were genotyped using the China Chip-1 Porcine SNP50K BeadChip. Genotype imputation was then performed with Beagle (v 5.4) [12], using the reference panel established by Tong et al. [13], and variants with imputation quality scores (DR^2^) >0.85 were remained. Subsequent quality control was implemented using PLINK (v1.9) [14]. SNPs with the minor allele frequency ≤ 0.01 and missing genotype rate ≥ 0.01 were excluded. After quality control, a total of 8,206,050 high-quality SNPs were used for further analyses.

### Heritability estimation

Principal component analysis (PCA) was performed using PLINK (v1.9) based on quality-controlled SNP genotype data to investigate the genetic structure of each breed individually and of the combined-population. The SNP-based heritability was estimated for each population and combined-population separately using GCTA (v 1.94.1) [15] based on the following model:

2 $${\mathrm{y} = Xb + g + \varepsilon ,}$$

where **y** represents the vector of phenotypes for the traits; **X** denotes the design matrices for fixed effects, including birth year and season, sex, age, and the top five principal components derived from the quality-controlled SNP genotype data to account for population structure; **b** denotes the vectors of fixed-effect coefficients; ${\bf{g}} \sim {\mathrm{N}}\left( {0,{\bf{G}}\sigma _{\mathrm{g}}^2} \right)$ denotes the vectors of random genetic effects, **G** is the genomic relationship matrix, calculate from SNP genotype data using GCTA with the –make-grm option; and ${\bf{\varepsilon }} \sim {\mathrm{N}}\left( {0,{\bf{I}}\sigma _{\mathrm{e}}^2} \right)$ denotes the vectors of residuals. The SNP-based heritability was then estimated as follows:

$${{\rm{h}}^2} = {{{\rm{\sigma }}_{\rm{g}}^2} \over {{\rm{\sigma }}_{\rm{g}}^2 + {\rm{\sigma }}_{\rm{e}}^2}}$$

where ${\rm{\sigma }}_{\rm{g}}^2$ denotes the additive genetic variance and ${\rm{\sigma }}_{\rm{e}}^2$ denotes the residual variance.

### Genome-wide association study

GWAS was performed for each population and for the combined-population using a linear mixed model implemented in GEMMA (v 0.98.1) [16]. The model followed the same structure as model (2), with an additional fixed-effect term for the SNP being tested:

3 $${\mathrm{y} = Xb + wa + g + \varepsilon ,}$$

where **w** represents the genotypes vector for the SNP; a is the additive effect of the SNP. To assess whether SNP associations with LMA and LMD were robust to adjustment for body weight (BW), additional sensitivity analyses were performed by including BW as a covariate in the GWAS models for LMA and LMD.

To account for the strict Bonferroni correction threshold (0.05/number of SNPs) [17], the effective number of independent SNPs was estimated using the SimpleM method [18] in R (v 3.5.1), yielding 420,894 independent SNPs. Accordingly, the thresholds for genome-wide significance and suggestive association were set at 1.2 × 10^−7^ (0.05/420,897) and 2 × 10^−6^ (1/420,897), respectively. Manhattan and Q-Q plots were produced using the CMplot package (v 0.1.4), and the haplotype block structure around the lead SNP was examined using LDBlockShow (v 1.40) [19].

### Epigenomic data analysis

Publicly accessible ATAC-seq and histone ChIP-seq datasets (H3K4me1, H3K4me3, H3K27ac, and H3K27me3) generated from adipose and skeletal muscle tissues of six-month-old male Yorkshire pigs were analyzed in this study [20]. Each assay included two biological replicates. Raw sequencing reads were quality-trimmed using Trim Galore v0.6.1 (https://www.bioinformatics.babraham.ac.uk/projects/trim_galore/) with default settings, followed by alignment to the Sus scrofa 11.1 reference genome using BWA v0.7.17 [21] in MEM mode. Reads with MAPQ below 30 were excluded using SAMtools v1.9 [22], and duplicate reads were removed using Picard v2.20.1 (Broad Institute. Picard Toolkit, http://broadinstitute.github.io/picard/). Then the normalized coverage tracks (BigWig files) were produced from the filtered BAM files using bamCoverage v3.5.1 [23] with CPM normalization. Replicate signals were averaged using wiggletools v1.2 [24] with the “mean” function, and the resulting bedGraph files were converted to BigWig format for visualization via bedGraphToBigWig v2.10 [25]. Footprint analysis was performed using TOBIAS v0.17.2 [26] with default parameters on narrow peaks called by MACS2 from merged biological ATAC replicates. Transcription factor (TF) motifs for vertebrates were obtained from the JASPAR CORE database [27]. Motif scanning in regions lacking ATAC peaks was conducted using the findMotifs.pl command of HOMER [28] to identify potential TF binding sites.

### HiChIP and snRNA-seq datasets

Processed chromatin interaction BED files from the H3K27ac HiChIP dataset of two-week-old male Yorkshire longissimus dorsi were downloaded from public repositories, with two biological replicates [29]. Visualization of epigenomic features and HiChIP interactions was performed with pyGenomeTracks v3.9 [30]. Normalized and quality-controlled snRNA-seq matrices of psoas major muscle (PMM) and subcutaneous adipose tissue (SAT) from 2-year-old female Bama pigs were obtained from the figshare repository [31]. Gene expression patterns were visualized in R using the Seurat package, and cell type-specific expression profiles of selected candidate genes were depicted as bubble plots generated with the DotPlot function.

### Dual-luciferase reporter assay

Pig genomic DNA was used as the template to amplify putative enhancer fragments encompassing the GWAS lead SNP. The amplified products were separated on a 1.5% agarose gel, purified, and then cloned into the modified pGL3-Promoter plasmids. Constructed plasmids were transfected to PSCs using Lipofectamine 3000, and incubated for 24 h with five technical replicates for each construct. Luciferase activity was measured by Dual-Luciferase Assay (YEASEN, 11402ES60) according to the manufacturer’s manual, and Renilla luciferase activity was normalized against Firefly luciferase activity.

### PheWAS and two-sample mendelian randomization

PheWAS results were obtained from the PigGTEx database [32]. Summary statistics for trait associations were extracted for each target gene and PheWAS plots were generated for visualization. Two-sample Mendelian randomization (MR) was performed using the TwoSampleMR package [33]. Muscle expression quantitative trait loci (eQTLs) from PigGTEx were used as exposures, and GWAS summary statistics of the trait of interest were used as outcome. Independent instrumental variables were selected based on association *p*-values (*p* < 1 × 10^−5^) and linkage disequilibrium clumping. Causal estimates were calculated using five MR methods.

## Results

### Summary statistics and heritability of meat yield traits

Phenotypic correlations among LMP, LMA, LMD, BFT, and BW were evaluated using Pearson’s correlation analysis (Additional file 1, Fig. S1). LMP showed a strong positive correlation with LMA (*r* = 0.78) and a moderate negative correlation with BFT (*r* = −0.47), whereas its correlation with BW was negligible (*r* = 0.03). Moderate positive correlations were observed between LMA and BW (*r* = 0.52), LMD and BW (*r* = 0.31), and BFT and BW (*r* = 0.40).

Descriptive statistics for LMP, LMA, and LMD in 4593 pigs are summarized in Table 1. Among the three breeds, Duroc pigs showed the highest LMP and LMA values but the lowest LMD compared with Yorkshire and Landrace pigs, consistent with previous study [4]. LMP showed the lowest phenotypic variability (CV = 3.09%-3.60%) and relatively higher heritability estimates (0.31–0.45), whereas LMD displayed the greatest variability (CV = 11.1%-12.7%) and the lowest heritability (0.22–0.28). Overall, heritability estimates for the three traits ranged from 0.21 to 0.45, with LMP and LMA exhibiting higher heritability in Landrace pigs and lower values in Duroc pigs. These results reflect substantial genetic contributions to variation in meat yield traits and highlight differences in the genetic architecture of muscle-related traits among commercial pig breeds.

**Table 1:** Descriptive statistics and heritability estimates for the three traits for three breeds and the combined population

Trait	Breed	Number	Mean ± SD	CV (%)	Min	Max	**h**^**2**^ **± SE**
LMP (%)	Yorkshire	2585	53.64 ± 1.68	3.14	48.42	67.95	0.40 ± 0.03
	Landrace	1290	53.95 ± 1.94	3.60	45.90	61.02	0.45 ± 0.04
	Duroc	718	55.61 ± 1.72	3.09	50.34	61.73	0.31 ± 0.07
	combined	4593	54.03 ± 1.89	3.50	45.90	67.95	0.41 ± 0.02
LMA (cm^2^)	Yorkshire	2585	37.58 ± 4.85	12.90	25.31	69.84	0.30 ± 0.03
	Landrace	1290	38.17 ± 4.79	12.54	25.73	54.90	0.37 ± 0.04
	Duroc	718	40.92 ± 4.71	11.51	26.95	61.03	0.21 ± 0.06
	combined	4593	38.27 ± 4.95	12.93	25.31	69.84	0.43 ± 0.02
LMD (mm)	Yorkshire	2585	51.47 ± 5.84	11.34	30.77	94.46	0.28 ± 0.07
	Landrace	1290	51.08 ± 5.69	11.14	26.27	71.98	0.22 ± 0.04
	Duroc	718	48.72 ± 6.19	12.71	21.54	75.50	0.22 ± 0.03
	combined	4593	49.81 ± 6.13	12.30	21.54	94.46	0.26 ± 0.02

LMP, lean meat percentage; LMA, longissimus muscle area; LMD, longissimus muscle depth. Mean ± SD indicates the mean value and standard deviation. CV (%) represents the coefficient of variation. h^2^ ± SE indicates the estimated heritability and its standard error

### Integrative functional characterization of LMP-associated QTLs and candidate genes

**Fig. 1:**
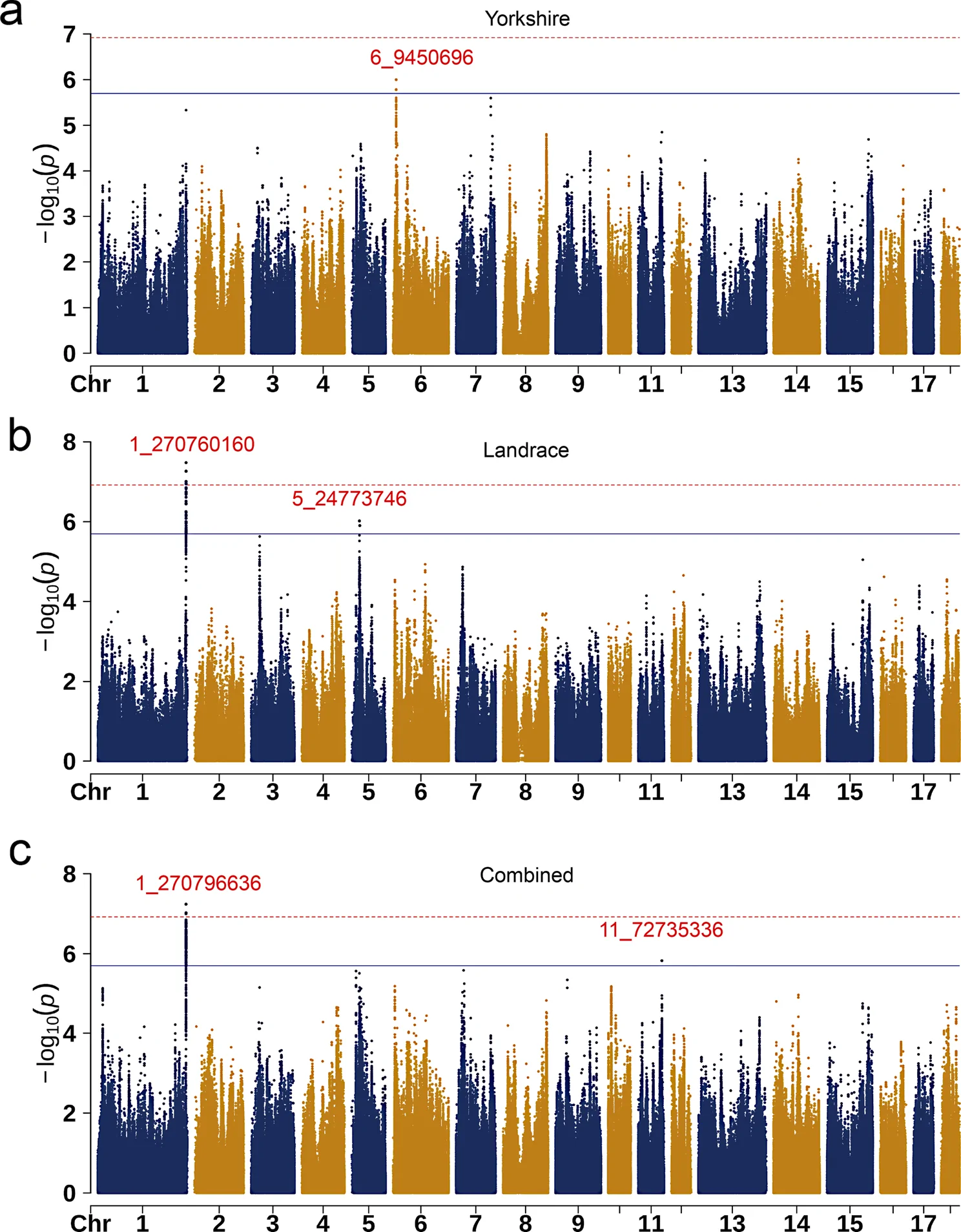
Genome-wide association results for lean meat percentage. Manhattan plots showing genome-wide association study results for lean meat percentage in (**a**) Yorkshire, (**b**) Landrace, and (**c**) combined-population. The red dashed horizontal line indicates the genome-wide significance threshold, whereas the blue solid line represents the suggestive significance threshold

The GWAS analysis for LMP identified 2, 130, and 155 SNPs reaching suggestive significance in the Yorkshire, Landrace, and combined-population, respectively (Fig. 1). No significant SNPs were detected in Duroc pigs, which may be attributable to the limit sample size. The genomic inflation factor (λGC) ranged from 0.94 to 1.01 (Additional file 2, Fig. S2a), indicating minimal inflation of test statistics and effective control of population stratification. The lead SNPs identified in different breeds were located on distinct chromosomes, suggesting breed-specific genetic backgrounds.

**Fig. 2:**
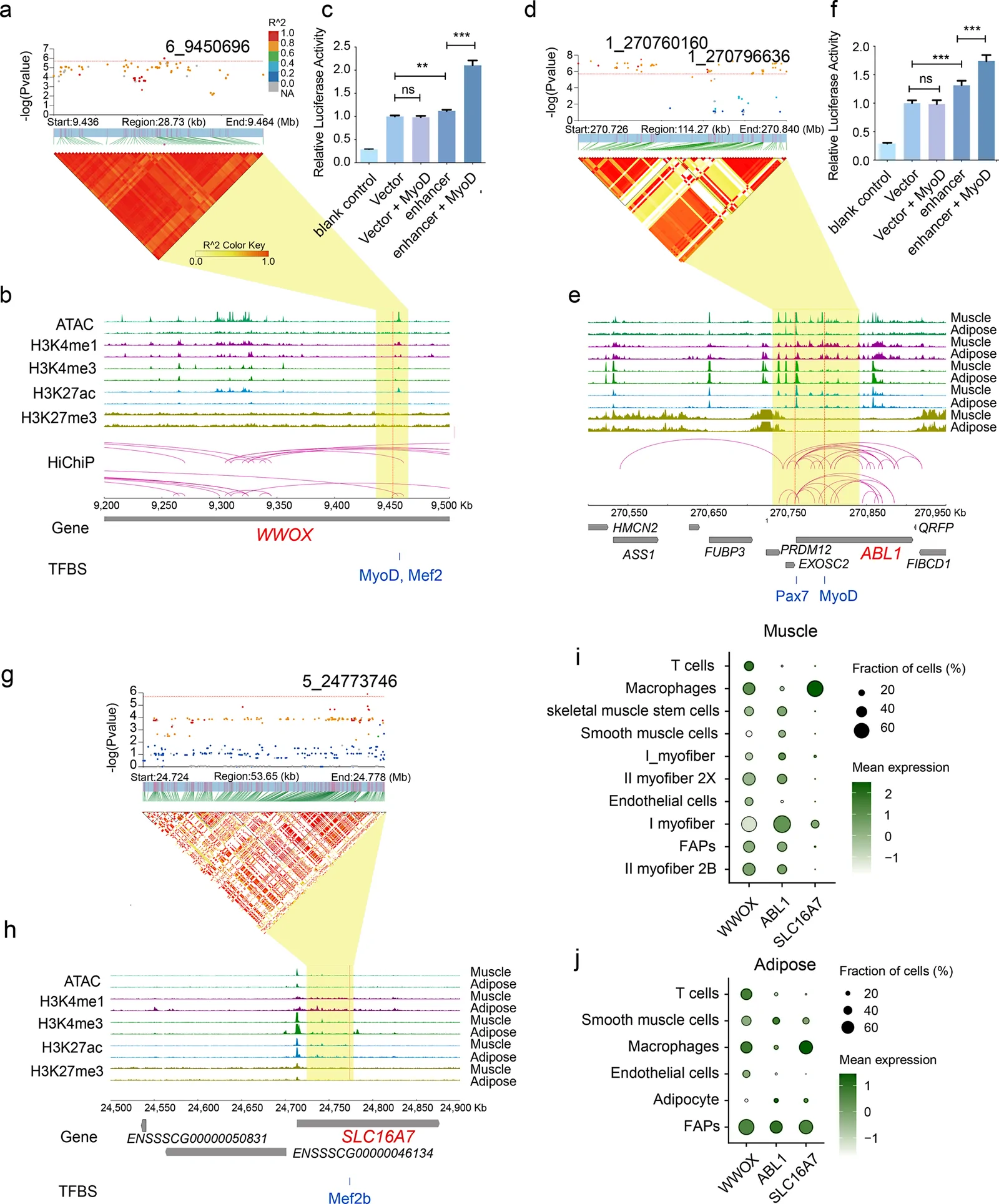
Epigenomic annotation and functional characterization of candidate quantitative trait loci associated with lean meat percentage. (**a, d, g**) linkage disequilibrium blocks surrounding the lead SNPs, the red horizontal line indicates the suggestive significance threshold of 2 × 10^−6^. (**b, e, h**) Epigenomic landscape of the candidate quantitative trait loci in muscle and adipose tissues. The candidate quantitative trait loci are highlighted by light green bars, and the positions of the lead SNPs are indicated by red vertical dashed lines. Predicted transcription factor binding motifs are highlighted in blue. (**c, f**) Dual-luciferase reporter assays validating the regulatory activity of putative enhancer elements. Relative luciferase activity is shown as mean ± standard deviation from three independent experiments. Statistical significance was determined using a two-tailed unpaired student’s t-test (**p < 0.01, ***p < 0.001). (**i, j**) Dot plots showing the expression patterns of candidate genes across muscle (**I**) and adipose (**j**) cell types based on snRNA-seq data

In Yorkshire pigs, the QTL (~28.73) surrounding the lead SNP 6_9450696 on SSC6 showed strong LD and was located within the genebody of *WWOX* (Fig. 2a–b). Notably, this QTL encompassed a putative enhancer element marked by H3K27ac, H3K4me1, and ATAC-seq signals in muscle tissue, which physically interacts with distal regulatory elements through chromatin looping. To explore the potential transcription factors involved in the regulation of *WWOX* expression, motif scanning was performed on the enhancer region, revealing a binding motif for the myogenic pioneer transcription factor MyoD. Consistently, footprint analysis identified binding motifs belonging to the Mef2 family within the same enhancer. Both MyoD and Mef2 are well-established regulators of skeletal muscle development and cooperate to form a classical myogenic regulatory network that synergistically activates muscle-specific promoters [34–36]. Notably, MyoD has also been shown to act as a pioneer architectural protein shaping lineage-specific 3D chromatin organization in skeletal muscle cells [37], which may contribute to the enhancer’s looping interactions with distal regulatory elements. Dual-luciferase reporter assays demonstrated that the putative enhancer region possesses transcriptional regulatory activity, which was significantly increased upon MyoD overexpression (Fig. 2c). These results provide supportive evidence that this genomic region may function as a regulatory element in muscle-related cellular contexts and suggest a potential regulatory relationship between the enhancer and the nearby candidate gene *WWOX*.

In Landrace pigs, we identified an LD block of approximately 114.27 kb on SSC1, which was also detected in the combined-population (Fig. 2d). The Landrace lead SNP 1_270760160 is located approximately 1.5 kb upstream of the active promoter region of the *ABL1*. Footprint analysis in muscle tissue identified a binding motif for the myogenic pioneer TF Pax7 at the *ABL1* promotor. In contrast, the lead SNP 1_270796636 identified in the combined-population is located within the genebody of *ABL1* and overlaps a putative enhancer marked by a broad H3K4me1 peak. Motif scanning in a 200-bp window centered on the lead SNP predicted binding motif of MyoD (Fig. 2e). Dual-luciferase reporter assays demonstrated the transcriptional regulatory activity of this putative enhancer element, and MyoD overexpression further enhanced its activity (Fig. 2f). Notably, this QTL represents a highly self-interacting chromatin domain enriched with multiple chromatin loops and activate histone modification signals, providing supportive evidence that the region may serve as an important regulatory element involved in muscle-related gene regulation. Another QTL (~53.7 kb) identified on SSC5 in Landrace pigs showed moderate LD and was located within the genebody of *SLC16A7* (Fig. 2g–h). Motif scanning predicted a putative binding motif for Mef2b.

To investigate the cell-type-specific expression patterns of candidate genes relevant to LMP, we analyzed publicly available snRNA-seq data from muscle and adipose tissues. In muscle tissue, *WWOX* and *ABL1* are broadly expressed in myofibers, skeletal muscle stem cells, and fibro/adipogenic progenitors (FAPs) (Fig. 2i), whereas in adipose tissue, all three candidate genes (*WWOX*, *ABL1*, and *SLC16A7*) showed relatively higher expression in FAPs (Fig. 2j). Notably, myofibers, muscle stem cells, and FAPs are key functional cell populations involved in skeletal muscle development, regeneration, and adipogenic regulation, suggesting that these candidate genes may play a relevant role in meat yield traits. In addition, PheWAS analyses provided supportive evidence for the prioritization of candidate genes, with *WWOX* showing a moderate association with LMD (*p* = 9.97 × 10^−4^), *ABL1* exhibiting its strongest association with meat-to-fat ratio (*p* = 1.96 × 10^−11^), and *SLC16A7* being most strongly associated with backfat thickness (*p* = 1.84 × 10^−7^) (Additional file 3, Fig. S3a-c).

We further applied MR analysis to explore the relationship between genetically predicted gene expression and the LMP trait. Due to the limited availability of instrumental SNPs in public databases, MR analysis could only be conducted for *ABL1*, for which six instrumental SNPs were available. The MR results showed a consistent positive direction of effect of *ABL1* gene expression on LMP across all five MR methods (Additional file 4, Fig S4a-b). However, none of the associations reached statistical significance (*p* > 0.05), likely reflecting limited statistical power due to the small number of instrumental variables. Sensitivity analyses showed no evidence of heterogeneity (Cochran’s Q test, *p* = 0.988) or directional horizontal pleiotropy (MR-Egger intercept test, *p* = 0.789). Therefore, these results should be interpreted as directional supportive evidence for candidate gene prioritization rather than evidence of a causal effect.

### Integrative functional characterization of LMA-associated QTLs and candidate genes

**Fig. 3:**
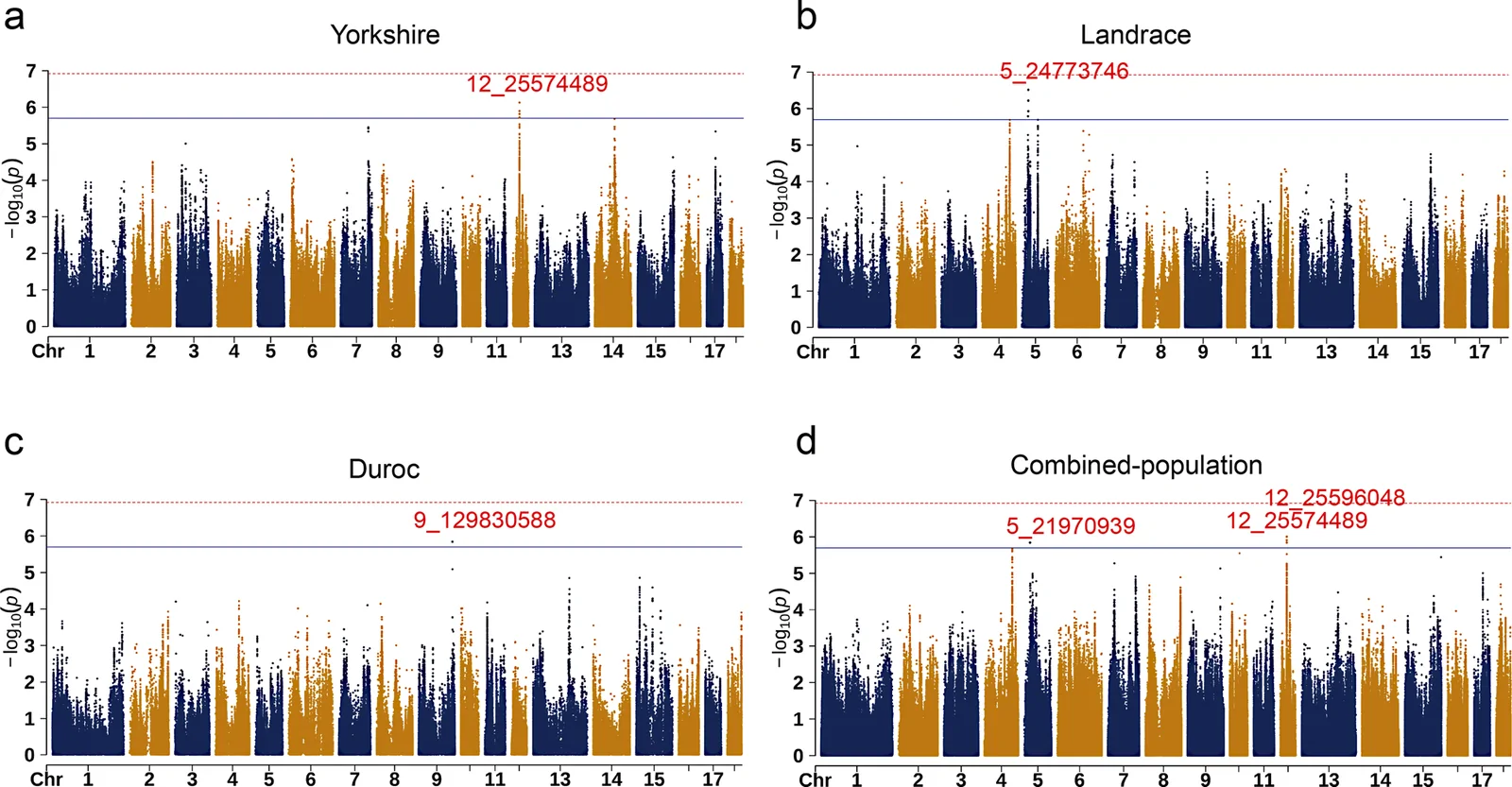
Genome-wide association results for longissimus muscle area. Manhattan plots showing genome-wide association study results for longissimus muscle area in (**a**) Yorkshire, (**b**) Landrace, (**c**) Duroc and (**d**) The combined-population. Significance thresholds are as described in Fig. 1 legend

For LMA, four, eight, one, and four SNPs reached suggestive significance for Yorkshire, Landrace, Duroc, and the combined-population, respectively (Fig. 3), λGC ranged from 0.97 to 1.04 (Additional file 2, Fig. S2b). Notably, the Landrace-specific lead SNP 5_24773746 was also identified in the LMP analysis described above and was located within the candidate gene *SLC16A7* (Fig. 2i–j), highlighting the potential importance of this locus in the regulation of muscle related traits in Landrace pigs.

**Fig. 4:**
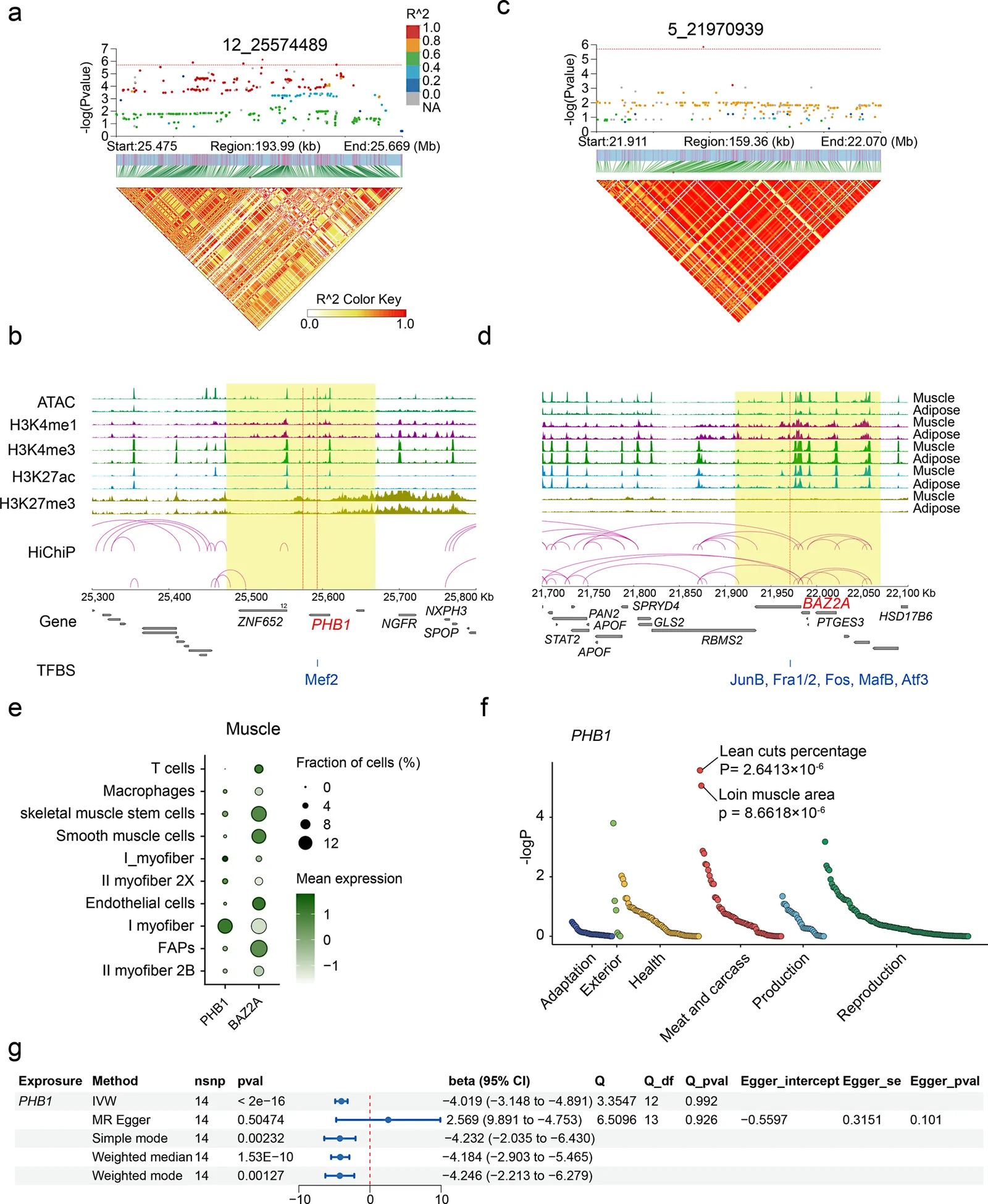
Epigenomic annotation and functional characterization of candidate quantitative trait loci associated with longissimus muscle area. (**a, c**) Linkage disequilibrium blocks surrounding the lead single nucleotide polymorphisms, (**b, d**) Epigenomic landscape of the candidate quantitative trait loci in muscle and adipose tissues. (**e**) Dot plot showing the expression patterns of candidate genes across muscle cell types based on snRNA-seq data. (**f**) Phenome-wide association study results for *PHB1*. (**g**) Forest plot of Mendelian randomization showing the estimated causal effect of *PHB1* expression in muscle on longissimus muscle area

A QTL spanning approximately 193 kb was identified on SSC12 in both the Yorkshire and combined-population (Fig. 4a). The Yorkshire lead SNP 12_25574489 overlapped a region enriched for H3K27me3, indicating localization within a polycomb-associated repressive chromatin domain and suggesting a potential silencer function (Fig. 4b). The combined-population lead SNP 12_25596048 mapped to the genebody of *PHB1*, Motif scanning in a 200-bp window centered on the lead SNP predicted binding motifs for Mef2 family transcription factors.

Another combined-population lead SNP 5_21970939 defined a QTL spanning approximately 159 kb and encompassing the candidate gene *BAZ2A* (Fig. 4c–d). Epigenomic profiling revealed that the *BAZ2A* promoter is marked by active histone modifications, including H3K4me3, H3K27ac, and H3K4me1, in both muscle and adipose tissues. Furthermore, multiple chromatin loops connect *BAZ2A* to distal regulatory elements in muscle, suggesting high transcriptional activity of this gene in these tissues. Motif scanning of lead SNP identified binding motifs for AP-1 family transcription factors, including JunB, Fos, MafB, and Atf3, suggesting potential regulation by AP-1-mediated transcriptional programs.

snRNA-seq of muscle tissue revealed that candidate gene *PHB1* is preferential expressed in type I (slow-twitch) myofibers, whereas *BAZ2A* exhibited broad expression across multiple muscle cell types (Fig. 4e). PheWAS further identified lean cut percentage and LMA as the top two traits associated with *PHB1* (Fig. 4f), and identified LMD as the top associated trait with *BAZ2A* among meat and carcass traits (*p* = 1.0 × 10^−5^), which is highly correlated with LMA (Additional file 3, Fig. S3d). Further MR analysis (14 instrumental SNPs) suggested a negative association between genetically predicted *PHB1* expression and LMA (Fig. 4g). Although the MR-Egger estimate showed an opposite effect direction, the IVW and other methods consistently supported a negative association. This discrepancy may reflect the lower statistical power and reduced precision of the MR-Egger method [38]. Sensitivity analyses showed no evidence of heterogeneity among instrumental variables (Cochran’s Q test, *p* = 0.992) or directional horizontal pleiotropy (MR-Egger intercept test, *p* = 0.101), supporting the robustness of the observed association. In addition, MR analysis suggested a positive association between genetically predicted *BAZ2A* expression and LMA. Although no evidence of heterogeneity (Cochran’s Q test, *p* = 0.961) or directional horizontal pleiotropy (MR-Egger intercept test, *p* = 0.994) was detected, the analysis was based on only three instrumental SNPs. Therefore, these results should be interpreted cautiously and are considered supportive evidence for prioritizing *BAZ2A* as a candidate gene associated with LMA. (Additional file 4, Fig. S4c-d).

### Integrative functional characterization of LMD-associated QTLs and candidate genes

**Fig. 5:**
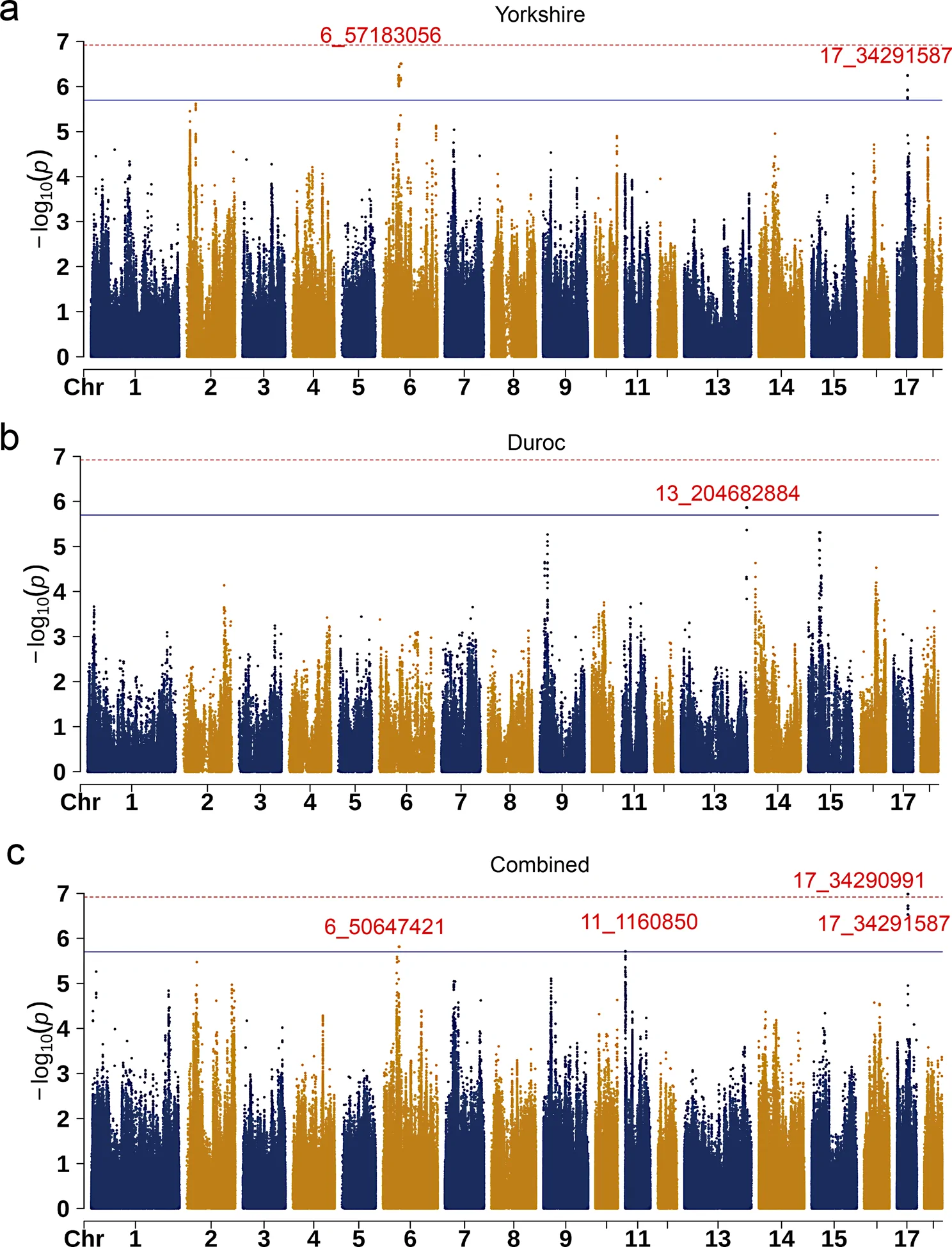
Genome-wide association results for longissimus muscle depth. Manhattan plots showing genome-wide association study results for longissimus muscle depth in (**a**) Yorkshire, (**b**) Duroc, and (**c**) The combined-population. Significance thresholds are as described in Fig. 1 legend

For LMD, 65, 3, and 9 SNPs reached suggestive significance threshold in Yorkshire, Duroc, and the combined-population, respectively, whereas no SNPs reached this threshold in Landrace pigs (Fig. 5). λGC ranged from 0.98 to 1.07 (Additional file 2, Fig. S2c).

**Fig. 6:**
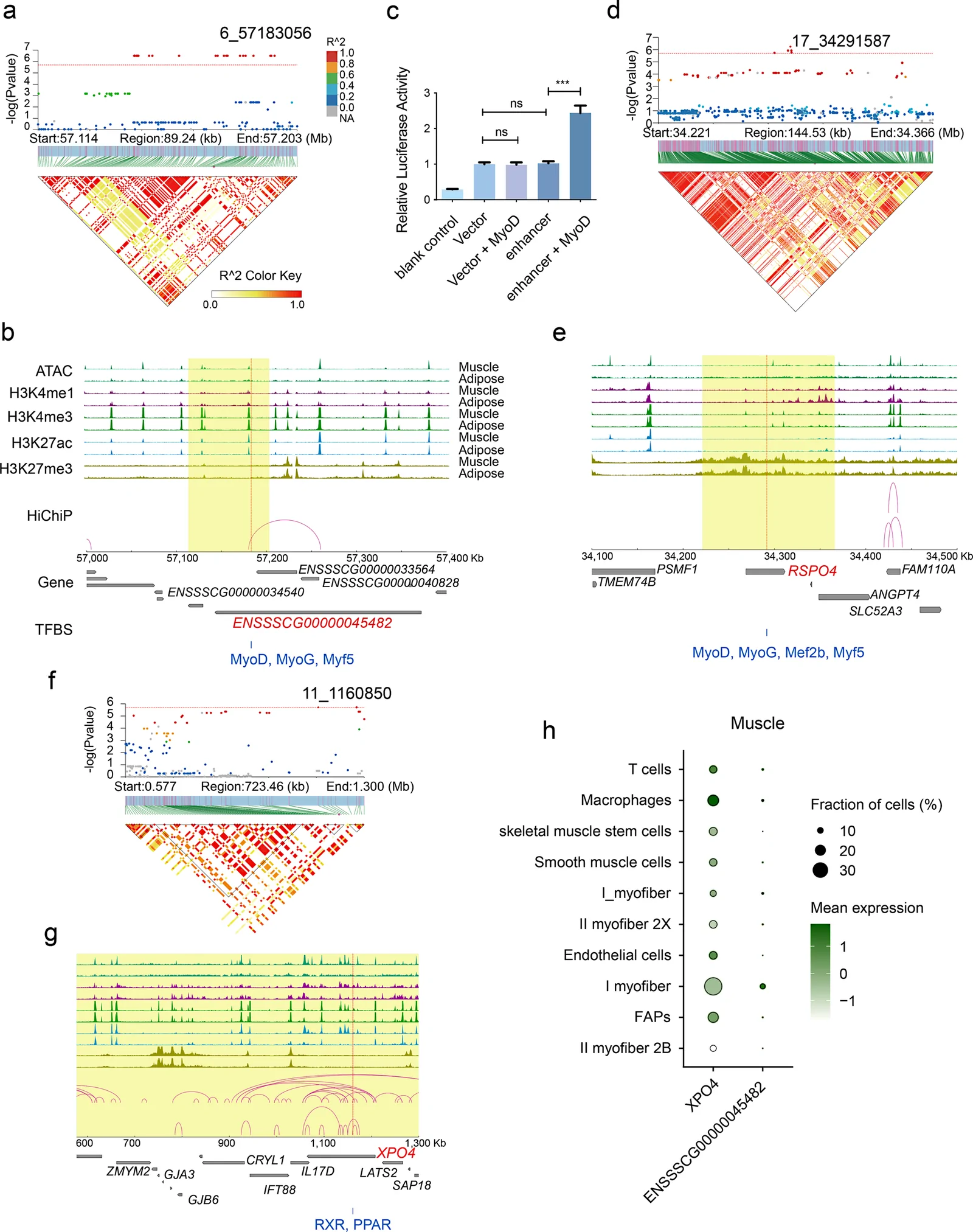
Epigenomic annotation and functional characterization of candidate quantitative trait loci associated with longissimus muscle depth. (**a, d, f**) linkage disequilibrium blocks surrounding the lead single nucleotide polymorphisms. (**b, e, g**) Epigenomic landscape of the candidate quantitative trait loci in muscle and adipose tissues. (**c**) Dual-luciferase reporter assays validating the regulatory activity of putative enhancer elements (***p < 0.001). (**h**) Dot plot showing the expression patterns of candidate genes across muscle cell types based on snRNA-seq data

The Yorkshire lead SNP 6_57183056 defined an 89.2 kb linkage block located within the gene body of *ENSSSCG00000045482* (Fig. 6a–b). A putative enhancer element was identified approximately 2 kb upstream of this SNP, which exhibited physical interaction with distal regulatory elements (Fig. 6b). Motif scanning revealed the binding motifs for key myogenic transcription factors, including MyoD, MyoG and Myf5, both surrounding the lead SNP locus and within the upstream enhancer region. Dual-luciferase reporter assays shows that overexpression of MyoD markedly increased the regulatory activity of the putative enhancer (Fig. 6c), providing supportive evidence that this regulatory element is responsive to myogenic transcription factors and supporting a potential role of *ENSSSCG00000045482* in muscle development. Another QTL (~144 Kb) encompassing the *RSPO4* gene on SSC17 was identified in both Yorkshire and the combined-population (Fig. 6d–e). Furthermore, motif scanning in a 200-bp window centered on the lead SNP revealed binding motifs for MyoD, MyoG, Mef2b and Myf5, indicating *RSPO4* may be involved in genetic regulation of myogenic processes.

In the combined-population analysis, the lead SNP 11_1160850 formed a large block spanning approximately 723 kb and map to the genebody of *XPO4* (Fig. 6f–g). Motif scanning of lead SNP revealed the binding motifs for RXR and members of the PPAR family, suggesting potential involvement of this locus in metabolic regulatory pathways.

snRNA-seq analysis of muscle tissue revealed that *XPO4* is broadly expressed across multiple muscle-related cell types and shows preferential expression in type I myofibers. In contrast, *ENSSSCG00000045482* exhibited generally low expression in muscle cells, with relatively higher expression detected only in type I myofibers (Fig. 6h). In parallel, PheWAS analysis revealed that LMD exhibited the strongest association with *XPO4* among meat and carcass traits (*p* = 7.1 × 10^−3^). *RSPO4* was also strongly associated with LMD (*p* = 4.5 × 10^−5^), whereas *ENSSSCG00000045482* showed a notable association with LMA (*p* = 4.2 × 10^−4^) (Additional file 3, Fig. S3e-g). These associations further support the potential involvement of these genes in muscle-related traits and carcass composition.

### Effect of body weight adjustment on GWAS signals for LMA and LMD

To assess the potential confounding influence of growth traits, additional GWAS analyses were performed for LMA and LMD with BW included as a covariate. The association statistics of lead SNPs identified in the primary GWAS together with results from the BW-adjusted sensitivity analyses are summarized in Table 2. For LMA, all significant signals in Yorkshire and the combined-population were substantially attenuated or fell below the significance threshold after BW adjustment; however, the major QTL still retained consistent directional trends. In contrast, the association landscapes in Landrace and Duroc remained largely unaltered, with key signals maintaining their significance (Table 2, Additional file 5, Fig. S5a). For LMD, most signals in Yorkshire pigs diminished after BW adjustment, whereas the major QTL identified in Landrace and the combined-population analyses remained robustly detectable (Table 2, Additional file 5, Fig. S5a).

**Table 2:** Summary of lead SNPs associated with meat yield traits in the primary GWAS and after body weight adjustment

Trait	Lead SNP	Breed	EA	NEA	MAF	P	β	SE	*P* **(BW)**	β (BW)
LEP	6_9450696	Combined	CT	C	0.446	1.9e-04	0.16	0.04		
		Yorkshire	C	CT	0.368	1.0e-06	−0.24	0.05		
		Landrace	CT	C	0.216	3.7e-01	0.08	0.09		
		Duroc	CT	C	0.193	3.5e-01	−0.10	0.11		
	1_270760160	Combined	C	T	0.107	9.4e-06	−0.28	0.06		
		Yorkshire	C	T	0.021	3.5e-01	−0.15	0.16		
		Landrace	C	T	0.215	3.3e-08	−0.49	0.09		
		Duroc	C	T	0.222	4.4e-01	−0.08	0.10		
	5_24773746	Combined	T	C	0.221	5.2e-03	−0.14	0.05		
		Yorkshire	T	C	0.164	8.6e-01	0.01	0.07		
		Landrace	T	C	0.390	1.2e-06	−0.41	0.09		
		Duroc	T	C	0.118	1.8e-01	0.21	0.15		
	1_270796636	Combined	TG	T	0.316	4.5e-07	−0.26	0.05		
		Yorkshire	TG	T	0.157	1.7e-03	−0.20	0.06		
		Landrace	TG	T	0.294	1.2e-06	−0.41	0.08		
		Duroc	T	TG	0.074	9.8e-01	−0.005	0.16		
LMA	12_25574489	Combined	A	G	0.259	1.4e-06	−0.65	0.13		
		Yorkshire	A	G	0.433	7.4e-07	−0.67	0.13	4.5e-05	0.12
		Landrace	A	G	0.057	2.5e-01	−0.43	0.38		
		Duroc	\	\	\	\	\	\		
	5_24773746	Combined	T	C	0.221	1.1e-02	−0.32	0.13		
		Yorkshire	T	C	0.164	5.1e-01	0.12	0.18		
		Landrace	T	C	0.390	3.1e-07	−1.03	0.20	1.7e-06	0.18
		Duroc	T	C	0.118	5.5e-01	0.25	0.42		
	5_21970939	Combined	C	T	0.15	1.4e-06	0.84	0.17	1.9e-05	0.16
		Yorkshire	C	T	0.016	8.2e-01	0.12	0.54		
		Landrace	C	T	0.431	2.2e-05	0.87	0.20		
		Duroc	C	T	0.118	5.1e-02	0.76	0.39		
LMD	6_57183056	Combined	A	G	0.202	2.0e-03	0.62	0.20		
		Yorkshire	A	G	0.097	3.1e-07	1.51	0.30	2.9e-05	0.29
		Landrace	A	G	0.178	3.6e-02	0.65	0.31		
		Duroc	G	A	0.376	2.0e-01	0.48	0.37		
	17_34291587	Combined	A	G	0.119	2.9e-07	1.14	0.22	3.4e-07	0.21
		Yorkshire	A	G	0.172	1.7e-06	1.16	0.24		
		Landrace	A	G	0.082	7.0e-03	1.13	0.42		
		Duroc	\	\	\	\	\	\		
	11_1160850	Combined	A	G	0.250	2.0e-06	0.78	0.16	1.2e-06	0.16
		Yorkshire	A	G	0.397	1.2e-04	0.64	0.17		
		Landrace	A	G	0.082	1.3e-02	1.04	0.42		
		Duroc	\	\	\	\	\	\		

EA, effect allele; NEA, non-effect allele; MAF, minor allele frequency. β, Effect size; SE, Standard error; *P* (BW), BW-adjusted *p* value; β (BW), BW-adjusted β

Overall, while BW adjustment conditional analysis reduced the statistical significance of certain Yorkshire- and combined-population-specific signals, the majority of the major QTL remained consistent, indicating that the primary GWAS findings are robust and fundamentally reflective of the genetic architecture of LMA and LMD. These findings also suggest that the detected associations capture both growth-mediated pleiotropic variations and muscle-specific genetic effects.

## Discussion

In this study, we conducted a comprehensive genome-wide and integrative functional genomic analysis to dissect the genetic and regulatory basis of meat yield-related traits in commercial pig populations. By combining GWAS across three major commercial breeds with epigenomic profiling, chromatin interaction data, transcription factor motif analysis, and multiple layers of functional prioritization, we identified several putative regulatory QTLs and biologically relevant candidate genes associated with LMP, LMA, and LMD.

### Functional relevance of prioritized candidate genes for LMP

Based on integrative functional genomics evidence and multi-layered post-GWAS analyses, *WWOX* was prioritized as a potential functional candidate gene influencing LMP in Yorkshire pigs, whereas *ABL1* and *SLC16A7* were identified as candidate genes in Landrace pigs. *WWOX* has been implicated in multiple metabolic processes, including fatty acid biosynthesis, cholesterol homeostasis, and triglyceride metabolism, which are integral to intramuscular fatty acid composition [39]. Consistently, previous studies have linked *WWOX* to economically important meat traits in cattle, including backfat thickness [40], loin eye area [41], subcutaneous fat thickness [42], and meat color [43], suggesting a conserved role in regulating carcass composition and meat quality. *ABL1* encodes a non-receptor tyrosine kinase activated by growth factors and regulating actin cytoskeleton remodeling, interacting with filamentous actin to modulate cell polarity and motility [44, 45]. The most significant QTL identified in both Landrace and the combined-population GWAS analyses encompasses *ABL1*, which has also been reported as a candidate gene for backfat thickness in pigs [46], supporting its potential role in regulating LMP. *SLC16A7* encodes a high-affinity monocarboxylate transporter expressed in tissues that rely on substrate uptake for energy metabolism, including skeletal muscle, liver, adipose tissue, and brain [47, 48]. Acting as a metabolic fuel sensor, *SLC16A7* expression responds dynamically to nutritional and metabolic status, such as fasting or obesity [48], and genetic variants in this gene have been associated with high-density lipoprotein cholesterol levels [49]. Accordingly, *SLC16A7* may contribute to the regulation of LMP and related meat quality traits by modulating energy substrate utilization and lipid metabolic processes, thereby influencing the balance between fat deposition and muscle energy metabolism. Overall, these genes likely shape LMP through effects on energy metabolism and lipid handling, highlighting their potential contribution to meat yield traits in pigs.

### Functional relevance of prioritized candidate genes for LMA

We prioritized *PHB1* in Yorkshire pigs, *SLC16A7* in Landrace pigs, where it was also associated with LMP, and *BAZ2A* in the combined-population as candidate genes potentially contributing to variation in LMA. The preferential expression of *PHB1* in type I myofibers is consistent with its role in maintaining mitochondrial integrity and supporting oxidative phosphorylation and cellular energy metabolism [50], as type I myofibers rely heavily on mitochondrial function to sustain high levels of oxidative metabolism [51]. In addition, the predicted Mef2 family transcription factors in the *PHB1* genebody are known to cooperate with PGC-1α to promote mitochondrial biogenesis and control type I fiber-selective gene expression [52], potentially including *PHB1* identified in this study. In addition to maintaining mitochondrial integrity, *PHB1* possesses intrinsic anti-proliferative and growth-suppressive properties [53]. Given the negative association between *PHB1* and LMA observed in the MR analysis, we hypothesize that *PHB1* may negatively regulate LMA by constraining the hypertrophic capacity of muscle fibers through its anti-proliferative and growth-suppressive functions. In postnatal muscle, fiber number is largely fixed, and muscle growth depends primarily on fiber hypertrophy supported by satellite cell activation and nuclear accretion [54]. Elevated *PHB1* expression may limit these processes, thereby reducing fiber hypertrophy and contributing to lower LMA. For *BAZ2A*, MR analysis indicated a positive association between genetically predicted *BAZ2A* expression and LMA. Consistent with this observation, predicted binding motifs for AP-1 family transcription factors suggest that *BAZ2A* may participate in AP-1-mediated transcriptional programs involved in muscle differentiation, remodeling, and growth-related signaling [55]. Notably, JunB, a key AP-1 component, has been shown to induces muscle hypertrophy and inhibit muscle atrophy [56]. Collectively, these findings support a model in which AP-1-dependent regulation of *BAZ2A* may contribute to muscle growth and increased LMA through pro-hypertrophic transcriptional programs. In contrast, *PHB1* may negatively regulate LMA through its anti-proliferative and growth-suppressive functions.

### Functional relevance of prioritized candidate genes for LMD

We identified *ENSSSCG00000045482* and *RSPO4* as key genes influencing LMD in Yorkshire pigs, and *XPO4* in combined-population. *ENSSSCG00000045482* is an uncharacterized protein-coding gene, dual-luciferase reporter assays demonstrated that a putative enhancer in its vicinity responds to MyoD overexpression, suggesting that this gene may be regulated by myogenic transcription factors and potentially participate in pathways influencing LMD. *RSPO4* encodes a member of the R-spondin family, which functions as a potent enhancer of the Wnt/β-catenin signaling pathway [57, 58]. Activation of this pathway has been shown to promote myoblast self-renewal, proliferation, and differentiation [59, 60]. Consistent with the potential role in myogenesis, overexpression of *DLK2* during the proliferative phase of ovine myoblasts resulted in increased *RSPO4* mRNA levels along with upregulation of other Wnt/β-catenin pathway genes [61], suggesting that *RSPO4* may contribute to myoblast proliferation. *XPO4* is predominantly expressed in type I muscle fibers. The presence of RXR and PPAR binding motifs within its genebody suggests that *XPO4* may operate within a PPAR-RXR-associated transcriptional context. This signaling axis is known to play a central role in skeletal muscle metabolic homeostasis and promotes the formation of oxidative type I muscle fibers, and its dysregulation has been implicated in muscle dysfunction and atrophy [62]. Consistent with this regulatory context, *XPO4* has previously been reported as a differentially expressed gene in a rat model of skeletal muscle atrophy [63], suggesting that *XPO4* may influence LMD through regulatory mechanisms responsive to the PPAR-RXR axis. Oveall, *RSPO4* may contribute to LMD by facilitating myoblast proliferation through activation of the Wnt/β-catenin signaling pathway, whereas *XPO4* may be involved in the maintenance of muscle hypertrophy by responding to the PPAR-RXR signaling axis.

### Limitations and future perspectives

Several limitations of this study should also be considered. First, TF binding was inferred from footprint and motif scanning analyses, which predict potential regulatory interactions but do not confirm direct binding in vivo. Experimental validation using TF-specific chromatin profiling will be required. Second, although the dual-luciferase reporter assays supported the regulatory potential of genomic regions containing the lead SNPs, allele-specific assays were not performed. Therefore, the functional effects of the associated variants require further validation. Third, because LMP is estimated from LMA, BFT, and BW, the strong correlation between LMP and LMA (*r* = 0.78), as well as the shared association signal at 5_24773746, may be partly attributable to the mathematical relationship among these traits. Therefore, the overlap between LMP and LMA associations should be interpreted cautiously. Fourth, the epigenomic, chromatin interaction, and single-nucleus RNA-seq data were derived from different individuals and developmental stages, which may introduce heterogeneity in regulatory inference. Future studies integrating matched multi-omics data from the same samples will improve regulatory resolution. Finally, although multiple lines of evidence were used to prioritize high-confidence candidate genes and QTLs, their functional roles have not yet been experimentally validated, warranting further in vitro and in vivo studies.

## Conclusions

In summary, this study combined genome-wide association analyses with integrative functional genomic approaches to investigate the genetic and regulatory basis of meat yield traits in commercial pig populations. We identified multiple trait-associated loci and putative regulatory QTLs associated with LMP, LMA, LMD, and prioritized candidate genes using multiple lines of functional evidence, including snRNA-seq, pheWAS, and MR analysis. Specifically, *WWOX*, *ABL1*, and *SLC16A7* were prioritized for LMP, *PHB1* and *BAZ2A* for LMA, and *ENSSSCG00000045482*, *RSPO4*, and *XPO4* for LMD. These findings provide new insights into the genetic architecture and potential regulatory mechanisms contributing to variation in meat yield traits and offer a valuable resource for future functional validation and precision breeding strategies in pigs.

## Electronic supplementary material

Below is the link to the electronic supplementary material.

Supplementary Material 1

Supplementary Material 2

Supplementary Material 3

Supplementary Material 4

Supplementary Material 5

Supplementary Material 6

## Data Availability

The data that support the findings of this study are available on request from the corresponding author bioducklily@hotmail.com, upon reasonable request. The publicly available ATAC-seq and histone ChIP-seq dataset can be accessed from the NCBI Gene Expression Omnibus (GEO) under accession number GSE158430. while the H3K27ac HiChIP dataset is available under accession number GSE189397. The publicly snRNA- seq Seurat objects of PMM and SAT were obtained from figshare (https://doi.org/10.6084/m9.figshare.c.7247752).
