# Supplementary material for: Genetic and regulatory architecture of meat yield traits in commercial pig populations
Source: Genet Sel Evol. 2026 Nov 9;58:82. doi: 10.1186/s12711-026-01104-2 (PMC13746421; doi:10.1186/s12711-026-01104-2)
Supplement: Supplementary file 1 [file 12711_2026_1104_MOESM1_ESM.pdf]

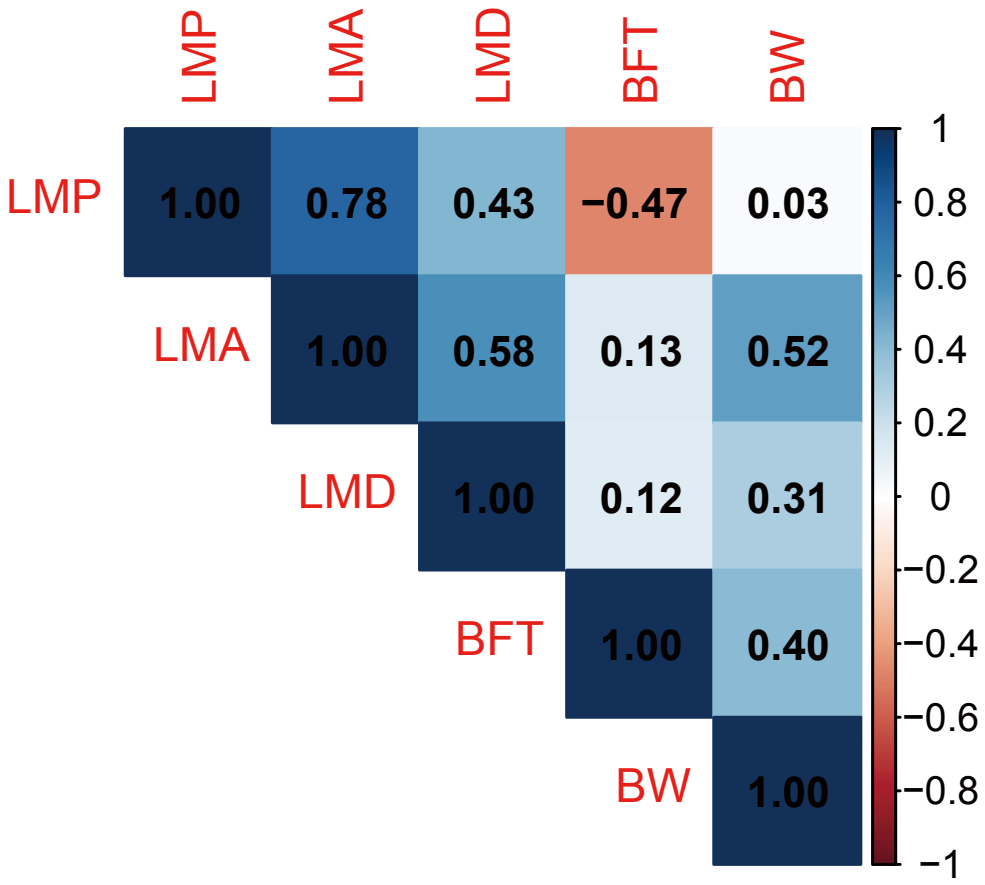
LMP
LMA
LMD
BFT
BW
1.00
0.78
0.43
−0.47
0.03
1.00
0.58
0.13
0.52
1.00
0.12
0.31
1.00
0.40
1.00
1
0.8
0.6
0.4
0.2
0
−0.2
−0.4
−0.6
−0.8
−1
