# Supplementary material for: Genetic and regulatory architecture of meat yield traits in commercial pig populations
Source: Genet Sel Evol. 2026 Nov 9;58:82. doi: 10.1186/s12711-026-01104-2 (PMC13746421; doi:10.1186/s12711-026-01104-2)
Supplement: Supplementary file 2 [file 12711_2026_1104_MOESM2_ESM.pdf]

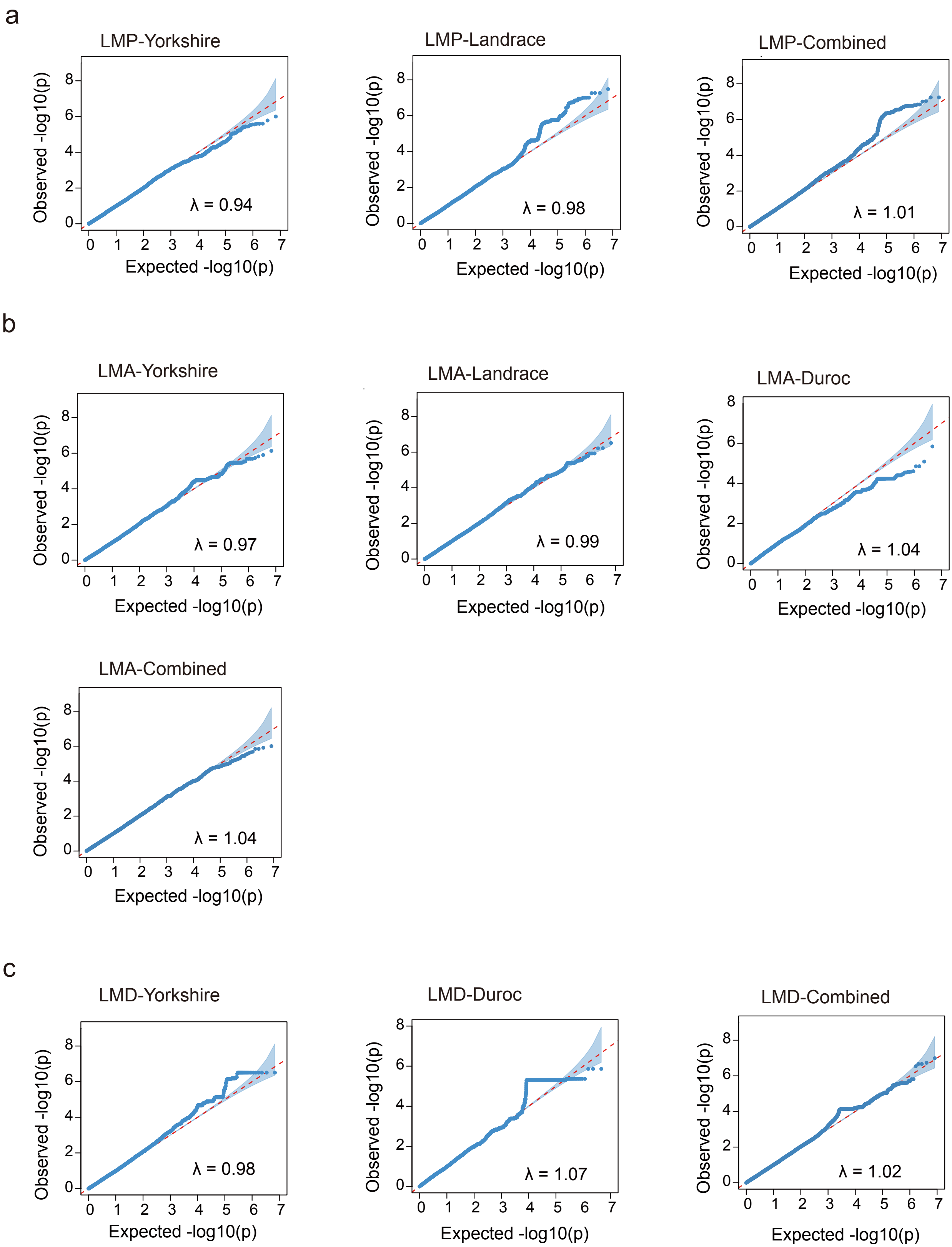
a
LMP-Yorkshire
λ = 0.94
LMP-Landrace
λ = 0.98
LMP-Combined
λ = 1.01
b
LMA-Yorkshire
λ = 0.97
LMA-Landrace
λ = 0.99
LMA-Duroc
λ = 1.04
LMA-Combined
λ = 1.04
c
LMD-Yorkshire
λ = 0.98
LMD-Duroc
λ = 1.07
LMD-Combined
λ = 1.02
Observed -log10(p)
Expected -log10(p)
0
1
2
3
4
5
6
7
8
