# Supplementary material for: Genetic and regulatory architecture of meat yield traits in commercial pig populations
Source: Genet Sel Evol. 2026 Nov 9;58:82. doi: 10.1186/s12711-026-01104-2 (PMC13746421; doi:10.1186/s12711-026-01104-2)
Supplement: Supplementary file 3 [file 12711_2026_1104_MOESM3_ESM.pdf]

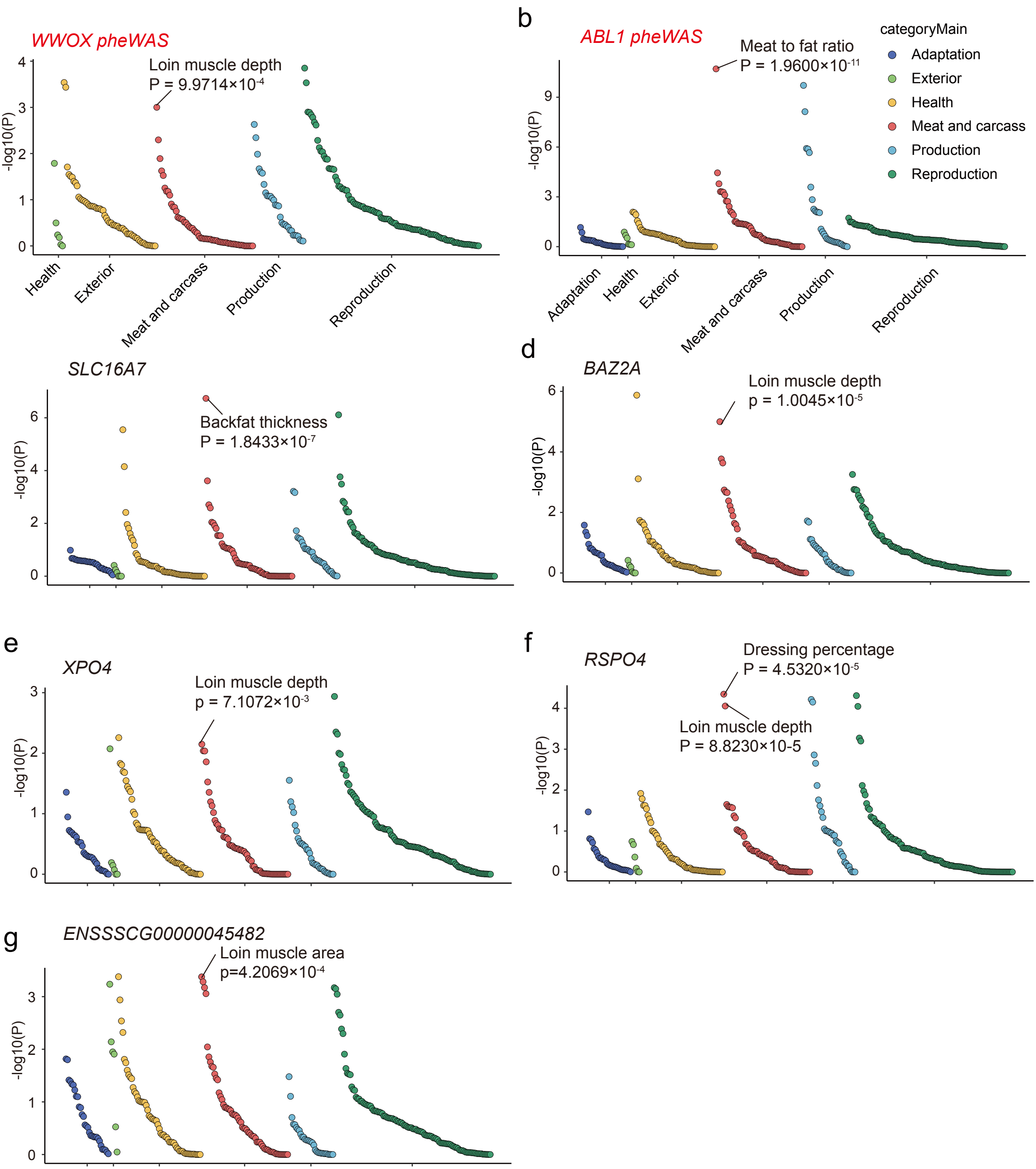
WWOX pheWAS
Loin muscle depth
P = 9.9714×10-4
-log10(P)
4
3
2
1
0
Health
Exterior
Meat and carcass
Production
Reproduction
b
ABL1 pheWAS
Meat to fat ratio
P = 1.9600×10-11
9
6
3
0
categoryMain
Adaptation
Exterior
Health
Meat and carcass
Production
Reproduction
Adaptation
SLC16A7
Backfat thickness
P = 1.8433×10-7
d
BAZ2A
Loin muscle depth
p = 1.0045×10-5
e
XPO4
Loin muscle depth
p = 7.1072×10-3
f
RSPO4
Dressing percentage
P = 4.5320×10-5
Loin muscle depth
P = 8.8230×10-5
g
ENSSSCG00000045482
Loin muscle area
p=4.2069×10-4
