# Supplementary material for: Genetic and regulatory architecture of meat yield traits in commercial pig populations
Source: Genet Sel Evol. 2026 Nov 9;58:82. doi: 10.1186/s12711-026-01104-2 (PMC13746421; doi:10.1186/s12711-026-01104-2)
Supplement: Supplementary file 4 [file 12711_2026_1104_MOESM4_ESM.pdf]

a

Exprosure	Method	nsnp	pval	beta (95% CI)	Q	Q_df	Q_pval	Egger_intercept	Egger_se	Egger_pval
ABL1	IVW	6	0.230	0.328 (−0.207 to 0.864)	0.3289	4	0.988			
	MR Egger	6	0.761	2.752 (−13.836 to 19.340)	0.411	5	0.995	−0.2084	0.7272	0.789
	Simple mode	6	0.623	0.246 (−0.675 to 1.168)						
	Weighted median	6	0.279	0.349 (−0.283 to 0.981)						
	Weighted mode	6	0.487	0.344 (−0.555 to 1.243)						

−4 0 4

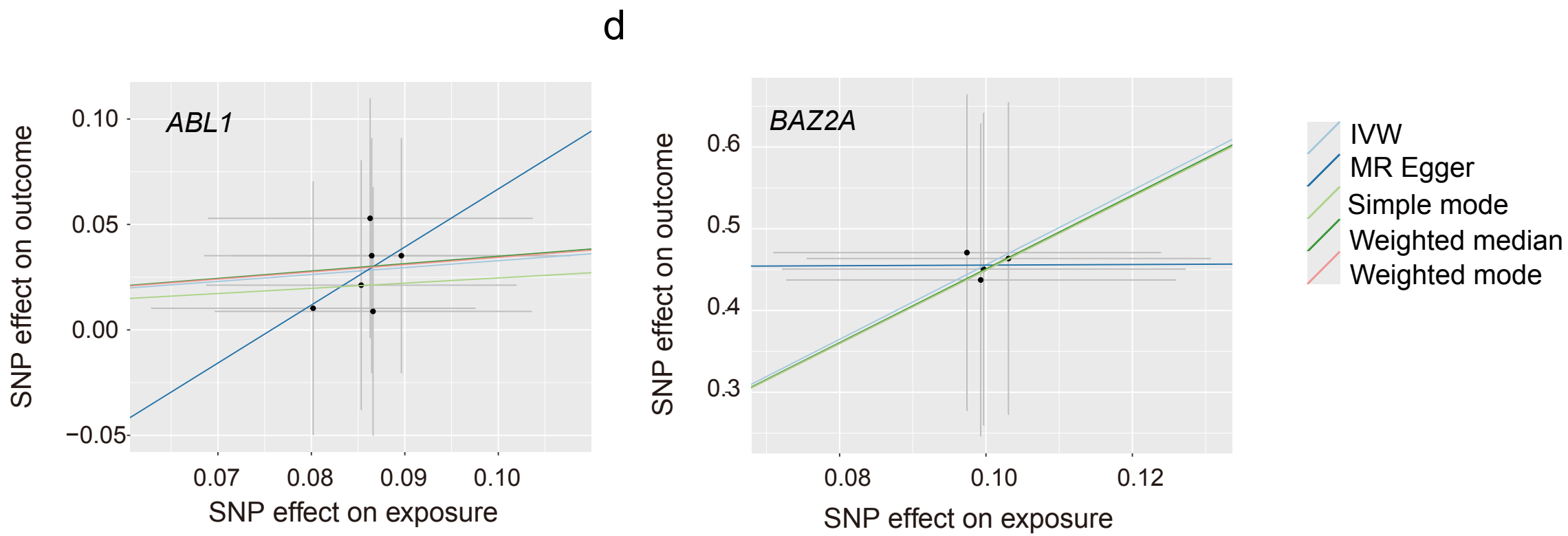

c

Exprosure	Method	nsnp	pval	beta (95% CI)	Q	Q_df	Q_pval	Egger_intercept	Egger_se	Egger_pval
BAZ2A	IVW	3	0.000052	4.480 (2.310 to 6.650)	0.0023	1	0.961			
	MR Egger	3	0.935660	4.097 (−75.100 to 83.300)	0.0024	2	0.999	0.0381	4.0247	0.994
	Simple mode	3	0.107530	4.515 (1.350 to 7.680)						
	Weighted median	3	0.007230	4.495 (1.210 to 7.770)						
	Weighted mode	3	0.121090	4.514 (1.120 to 7.910)						

−10 0 10
