# Supplementary material for: Genetic and regulatory architecture of meat yield traits in commercial pig populations
Source: Genet Sel Evol. 2026 Nov 9;58:82. doi: 10.1186/s12711-026-01104-2 (PMC13746421; doi:10.1186/s12711-026-01104-2)
Supplement: Supplementary file 5 [file 12711_2026_1104_MOESM5_ESM.pdf]

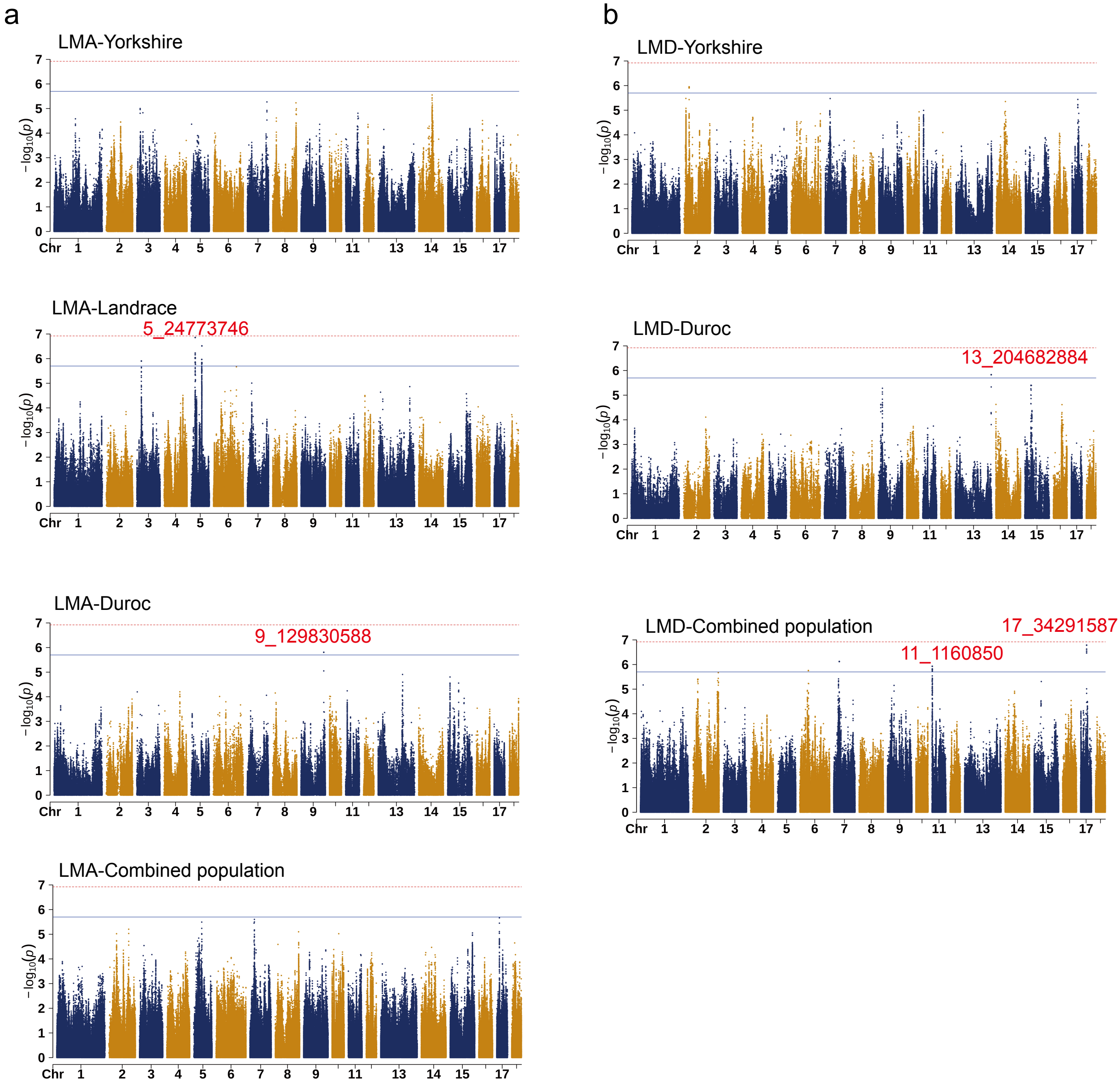

a
LMA-Yorkshire
LMA-Landrace
5_24773746
LMA-Duroc
9_129830588
LMA-Combined population
b
LMD-Yorkshire
LMD-Duroc
13_204682884
LMD-Combined population
17_34291587
11_1160850
−log10(p)
Chr 1 2 3 4 5 6 7 8 9 11 13 14 15 17
