# Supplementary material for: Genetic and regulatory architecture of meat yield traits in commercial pig populations
Source: Genet Sel Evol. 2026 Nov 9;58:82. doi: 10.1186/s12711-026-01104-2 (PMC13746421; doi:10.1186/s12711-026-01104-2)
Supplement: Supplementary file 6 [file 12711_2026_1104_MOESM6_ESM.docx]

**Additional files**

**Additional file 1: Figure S1**

Format: pdf

Title: Pearson correlation matrix among meat yield-related traits.

Description: Phenotypic correlations among lean meat percentage (LMP), longissimus muscle area (LMA), longissimus muscle depth (LMD), backfat thickness (BFT), and body weight (BW) were estimated using Pearson’s correlation coefficients.

**Additional file 2: Figure S2**

Format: pdf

Title: Quantile-quantile (Q-Q) plots of genome-wide association studies in pigs.

Description: Q-Q plots showing the distribution of observed versus expected −log₁₀(*P*) values for GWAS analyses of (a) lean meat percentage (LMP), (b) longissimus muscle area (LMA), and (c) longissimus muscle depth (LMD).

**Additional file 3: Figure S3**

Format: pdf

Title: Phenome-wide association study results for candidate genes associated with meat yield traits in pigs.

Description: (a-c) *WWOX* (a), *ABL1* (b), and *SLC16A7* (c) associated with lean meat percentage (LMP). (d) *BAZ2A* associated with longissimus muscle area (LMA). (e-g) *XPO4* (e), *RSPO4* (f), and *ENSSSCG00000045482* (g) associated with longissimus muscle depth (LMD).

**Additional file 4: Figure S4**

Format: pdf

Title: Mendelian randomization analyses for candidate genes associated with meat yield traits in pigs.

Description: (a) Forest plots showing the estimated causal effects of *ABL1* expression on lean meat percentage (LMP) trait. (b) Forest plots of *BAZ2A* expression on longissimus muscle area (LMA) trait. (c) Scatter plot illustrating the relationship between *ABL1* expression and LMP. Each point represents a single nucleotide polymorphism (SNP) used as an instrumental variable. (d) Scatter plot of *BAZ2A* expression vs. LMA

**Additional file 5: Figure S5**

Format: pdf

Title: Manhattan plots of genome-wide association analyses for longissimus muscle area and longissimus muscle depth with body weight included as a covariate

Description: Manhattan plots showing the results of genome-wide association analyses for (a) longissimus muscle area (LMA) and (b) longissimus muscle depth (LMD) after including body weight (BW) as a covariate in the GWAS model. Highlighted SNPs correspond to loci that overlap with association signals identified in the primary GWAS analyses without BW adjustment. The red dashed horizontal line indicates the genome-wide significance threshold, whereas the blue solid line represents the suggestive significance threshold.
